# Levels of Growth Factors and IgA in the Colostrum of Women from Burundi and Italy

**DOI:** 10.3390/nu10091216

**Published:** 2018-09-03

**Authors:** Daniel Munblit, Priya Abrol, Shreya Sheth, Li Yan Chow, Ekaterina Khaleva, Alan Asmanov, Silvana Lauriola, Ezio M. Padovani, Pasquale Comberiati, Attilio L. Boner, John O. Warner, Robert J. Boyle, Diego G. Peroni

**Affiliations:** 1 Department of Paediatrics, Imperial College London, London W2 1NY, UK; p.abrol@doctors.org.uk (P.A.); shreyasheth@me.com (S.S.); lychow8@gmail.com (L.Y.C.); j.o.warner@imperial.ac.uk (J.O.W.); r.boyle@nhs.net (R.J.B.); 2 Faculty of Pediatrics, Sechenov University, 119991 Moscow, Russia; 3 inVIVO Planetary Health, Group of the Worldwide Universities Network (WUN), 6010 Park Ave, West New York, NJ 07093, USA; 4 Department of Paediatrics, Saint-Petersburg State Paediatric Medical University, 194353 Saint-Petersburg, Russia; doctor.khaleva@gmail.com; 5 The Research and Clinical Institute for Pediatrics named after Academician Yuri Veltischev of the Pirogov Russian National Research Medical University, 125412 Moscow, Russia; alan-asmanov@yandex.ru; 6 Department of Life and Reproduction Sciences, Section of Paediatrics, University of Verona, 37124 Verona, Italy; silvana.lauriola@ospedaleuniverona.it (S.L.); ezio.padovani@univr.it (E.M.P.); pasquale.comberiati@gmail.com (P.C.); attilio.boner@univr.it (A.L.B.); 7 Department of Clinical and Experimental Medicine, Section of Paediatrics, University of Pisa, 56126 Pisa, Italy

**Keywords:** breast milk, human milk, colostrum, IgA, HGF, TGF-β, growth factors, geographical location

## Abstract

Colostrum is produced in the first days postpartum. It is a known source of immune mediators for a newborn within the first week of life. Although it is still unclear if colostrum composition varies between populations, recent data suggest differences. Hepatocyte growth factor (HGF); transforming growth factor-β (TGF-β) 1, 2, and 3; and immunoglobulin A (IgA) are key immunological components of colostrum that stimulate neonatal gastrointestinal and immune system development. We aimed to investigate the differences in the concentration between immune markers in the colostrum of mothers living in Burundi and Italy, and to identify the factors associated with differences. In this cross-sectional birth cohort study, a total of 99 colostrum samples from Burundian (*n* = 23) and Italian (*n* = 76) women were collected at 0 to 6 days postpartum. A clinical chemistry analyser was used for IgA quantification and electro-chemiluminescence, for HGF and TGFβ1-3 assessment. A univariate analysis and multivariate linear regression model were used for statistical testing. The concentrations of TGF-β2 (*p* = 0.01) and IgA (*p* < 0.01) were significantly higher in the colostrum from the women residing in Burundi than in Italy, both in a univariate analysis and upon the adjustment for confounding factors. A similar trend is seen for HGF, reaching statistical significance upon a multivariate analysis. We found a moderate to strong positive correlation between the TGF-β isoforms and IgA concentration in both countries (*p* < 0.01), with stronger concentration in the colostrum from Burundi. The results of this study are in support of previous data, suggesting that concentration of the immune active molecules is higher in the human milk of women residing in developing countries. However, with a small sample size, caution must be applied, as the findings require further confirmation. Future work should also be focused on other factors (e.g., lipid and microbial composition), as well as the investigation into colostrum and between populations comparison, adjusting for potential confounders.

## 1. Introduction

Human milk is a first source of nutrition for a newborn child and is globally accepted to be beneficial for the developing infant [1]. The advantages of breastfeeding include the transference of multiple immune factors, maturation of gut immunity, and anti-inflammatory effects [2]. The rising burden of non-communicable diseases increased an interest in their association with human milk (HM) cytokine composition [3,4]. It has been hypothesised that immune mediators in HM may play an important role in both the maturation of the newborn intestine and in stimulating immune system activation [5]. Previous prospective and longitudinal studies provided conflicting evidence on a breastfeeding protective effect on non-communicable [6] and communicable [7] outcomes, and have led to a suggestion that it may be due to the diversity of human milk composition between individuals [8].

Colostrum is the first human milk produced within the first days postpartum, and it allows for the transport of a high concentration of growth factors per unit volume. Hepatocyte growth factor (HGF) is secreted into human colostrum by multipotent mesenchymal stem cells [9]. HGF is expressed in BM as well as in the epithelial cells of the female reproductive tract and the gastrointestinal tract [10]. Patki et al. found greater concentrations of HGF in colostrum compared with paired umbilical cord serum samples [11]. It has been postulated that an elevated concentration of HGF in human milk is needed to maintain proliferation, angiogenesis, and intestinal tissue maturation through paracrine and endocrine signaling [9]. Additionally, HGF may regulate the proinflammatory vascular endothelial growth factor (VEGF) production from endothelial cells [12], and provide a complementary effect with VEGF on the neonatal gut [9]. 

Transforming growth factor-β (TGF-β) is an anti-inflammatory cytokine found in human colostrum. Throughout lactation, all three isoforms of the TGF-β superfamily (TGF-β1, TGF-β2, and TGF-β3) are produced, of which 95% is TGF-β2. The known effects of maternal TGF-β include oral and gut tolerance through the immunosuppressive action on neonatal T-lymphocytes [13,14]. Enteral exposures trigger neonatal antigen presenting cells in mucosal surfaces, to suppress inflammatory immune responses to common antigens via TGF-β2 mediated tolerance [15], which may lead to the inhibition of antigen specific T-lymphocytes’ proliferation [16]. It has been shown that intact cow’s milk protein (bovine alpha-S1 casein) is found in human colostrum, and a failure to tolerate this antigen could lead to a sensitisation for cow’s milk protein in the infant [17]. Some authors suggest that the TGF-β presence in colostrum is of a particular importance, as it may be partially responsible for the control of inflammatory processes that could lead to atopic sensitisation [18,19,20]. 

TGF-β is also responsible for the antibodies produced by B-lymphocytes’ class-switching, in particular, secretory immunoglobulin A (IgA). Japanese researchers found a correlation between the TGF-β1 concentration in human milk and IgA levels in the infant serum [21]. IgA secretion in human milk is vital to provide passive immune protection, as the newborn infant is unable to synthesise antibodies until 30 days postpartum [22]. Therefore, TGF-β mediated tolerance and IgA antibody synthesis are both key components for infant immune development. 

Previous studies have highlighted the importance of maternal and environmental factors’ influence on BM composition. These include both genetic and environmental factors, such as diet, psychological background, and maternal atopic status [23,24,25,26]. Very few studies have assessed the levels of immune mediators in the human milk of women from Africa and Europe; Holmlund and colleagues identified that Swedish mothers born in Mali retained higher breast milk TGF-β concentrations in comparison to women of Swedish origin [27]. The difference in association between the level of TGF-β in colostrum and ethnicity was supported further by Aihara Y. et al., explaining it by variations in the consumption of animal protein and the mode of delivery of lactating mothers in Japan and Nepal [28]. Recently, Ruiz and Espinosa-Martos et al. assessed the human milk immunological composition of women residing in different geographical locations, including Europe and Africa, showing a substantial variation within and, particularly among, human subpopulations [29].

Based on the The United Nations Children’s Fund (UNICEF) reported childhood mortality rate under the age of five, in Italy, it is 4 per 1000, whereas in Burundi, the rate is 104 per 1000, with 52% of these deaths being attributable to fatal childhood infections [30,31]. We hypothesised that the levels of immune active components, which may be able to reduce neonatal infection risk, in colostrum will be higher in the colostrum of Burundi women. Evidence suggests that not only bacterial exposures, but also a large range of other maternal and environmental factors, may be attributed to changes in the growth factors and IgA concentration [32], subsequently impacting the risk of non-communicable diseases [33] or infection [34] development. 

The aim of the study was to investigate the levels of all of the TGF-β isoforms (TGF-β1, 2, and 3), HGF, and IgA in the colostrum samples from Italy and Burundi, and to assess the potential factors responsible for the differences.

## 2. Materials and Methods 

### 2.1. Design

This cross-sectional birth cohort study involved mother–newborn pairs recruited from postnatal wards. The inclusion criteria for the study were healthy term infants and their mothers willing to comply with the study procedures. The exclusion criteria were maternal immunosuppressive treatment during lactation, or severe illness; infants with a major birth defect, admitted to neonatal intensive care; other severe illnesses; and inability to express colostrum. 

Informed consent was obtained from all of the participants who volunteered to take part, along with a colostrum sample and a questionnaire. 

The investigations and sample collection were conducted following ethical approval by the ethics committees in the two countries participating in this study, the Ethical Committee of the Azienda Ospedaliera di Verona (Italy) (approval N°1288) and the Ngozi Burundi Hospital approval, which was in accordance with Italian standards. 

### 2.2. Setting

The women were approached at postnatal wards in two very different environments, namely: in a developed country, G.B. Rossi Hospital, Verona, Italy, and in a developing country, the Hospital of Ngozi, Republic of Burundi. All of the samples were collected in the hospital by a highly skilled member of a research team. The clinical data on the participating infants and mothers were extracted from the hospital notes.

### 2.3. Colostrum Samples Collection

The participants were given sterile tubes to collect their own colostrum (once, in the first six days of life). Instructions were given for the collection of samples by manual expression or by collecting the drip from the contralateral breast during feeding. The colostrum samples were frozen at −20 °C in Burundi and −50 °C in Verona, within 12 h of collection, until their transfer to London. All of the samples were transported to Imperial College London, at −70 °C, where the samples were stored at −80 °C until analysis. All of the samples were analysed at Imperial College London facilities to ensure standardisation. After thawing, the samples were centrifuged at 1500× *g* for 15 min at 4 °C. The lipid layer was removed with a pipette tip, and the aqueous fraction was analysed for immune modulators.

### 2.4. Immune Mediators Measurement

We used electro-chemiluminescence to measure the immune mediators in the colostrum samples, for HGF and TGFβ1-3 (MesoScale Discovery, Rockville, MD, USA). LThe laboratory experiments were described in detail elsewhere [26]. In brief, the samples were run in duplicate, according to the manufacturer’s protocol, using an 8-point standard curve. No dilution was used for the HGF, and a 1:2 dilution was used for the TGFβ assays, following pilot experiments, which showed that the TGFβ2 level in the undiluted milk samples was often greater than the upper limit of detection. 

Using an Abbott Architect clinical chemistry analyser^®^ (Abbott, Abbott Park, IL, USA.), the IgA concentration analysis was conducted as described earlier [35]. The colostrum samples were centrifuged at 3000× *g* for 15 min at 4 °C to remove the lipaemic interference on analysis. Then, 300 μL of the supernatant was transferred into the cuvettes using pipettes. The samples were diluted to 1:5 for the IgA quantification. The IgA analysis determined the immunoturbidity of the immune insoluble complexes formed in the presence of the reagent. The IgA analysis with the corresponding reagents was performed according to the manufacturer’s protocol. 

### 2.5. Data Analysis

Based on previously published research statistical power calculations [36], the sample size of *n* = 48 in the Italy group and *n* = 22 in Burundi group allows for 80% power at a 0.05 significance level, which would allow for detecting a 34% difference between the groups for IgA and 36% for TGF-β in the colostrum. For the purpose of this study, only those samples with all data points available were included in the statistical analysis. The differences between the concentrations of HGF; TGF-β1, 2, and 3; and IgA in the Burundian and Italian colostrum samples were calculated with GraphPad Prism, Version 6.0 (GraphPad Software, La Jolla, CA, USA), for parametric data, using an unpaired *t*-test, Pearson’s chi-squared test, and Fisher’s exact test. The Mann–Whitney U test and Kruskal–Wallis one-way analysis of variance (ANOVA) were used for the non-parametric data. The correlations between TGF-β1, 2, and 3 with IgA were determined by Spearman’s rank correlation coefficient. The multivariate linear regression using a backward stepwise entry, was conducted using IBM SPSS Statistics software for Windows, Version 20 (IBM Corp, Armonk, NY, USA). The factors in the regression model included the maternal age, country of origin, animal contact, and parity. The results were considered significant when the *p*-values were reported at a level less than 0.05.

## 3. Results

### 3.1. Study Participants Demographics

Based on the questionnaire completed by all of the participants in the study, the demographic characteristics of the cohort have been assessed. Out of the total 117 women providing colostrum (Italy, *n* = 76; Burundi, *n* = 41), the full demographic details were available for 99 mothers only, and were included in the analysis (Italy, *n* = 76; Burundi, *n* = 23). Their demographic data is shown in Table 1. The maternal and parturition characteristics were different between the samples from Burundi and Italy. The women in Burundi were younger than the women in Italy (*p* < 0.01) and had more pregnancies (*p* < 0.01). The infants from Burundi were characterised by a lower weight and gestational age (*p* < 0.01 for both).

### 3.2. Quantification of Factors in Colostrum Samples

#### 3.2.1. Univariate Analysis Results

**Figure 1:**
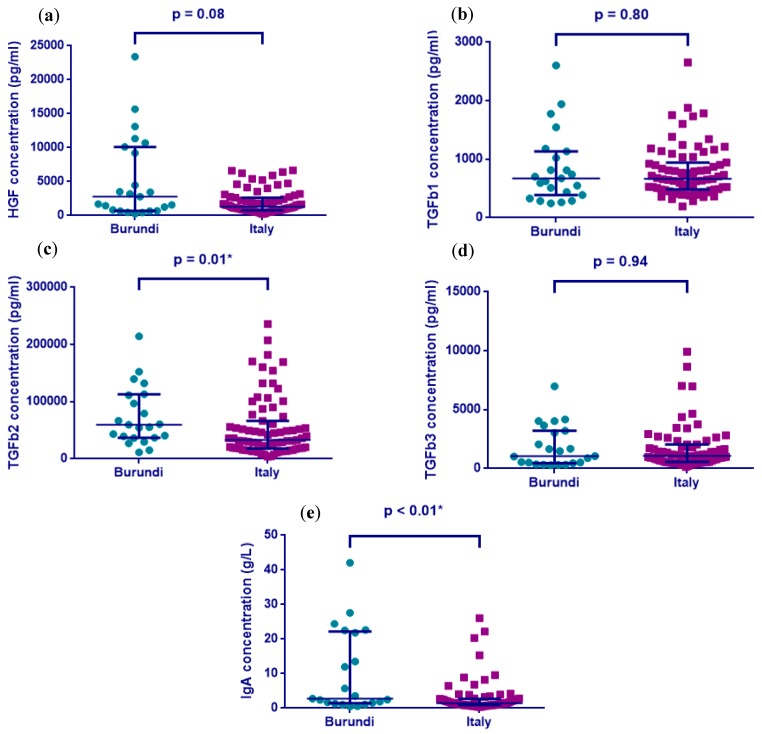
Comparison of growth factors: (**a**) hepatocyte growth factor (HGF), (**b**) transforming growth factor-β1 (TGF-β1), (**c**) TGF-β2, (**d**) TGF-β3, and (**e**) immunoglobulin A (IgA) raw concentrations in the colostrum samples from Burundi and Italy, using the Mann–Whitney U test. The results were considered significant when the *p*-values were reported at a level less than 0.05 *.

The analysis of the TGF-β1 and TGF-β3 raw concentrations found no significant difference between the Burundian and Italian women. The HGF levels did not differ significantly, but there was a trend for higher levels in the colostrum of the Burundian women (2785 pg/mL and 697.4–10,107 vs. 1316, 785–2628, for the median and interquartile range (IQR), respectively, *p* = 0.08) (Figure 1a). The TGF-β2 concentrations in the Burundi women’s colostrum were significantly higher than the colostrum samples from the Italian women (59,708 and 36,865–113,221 pg/mL vs. 33,176 and 18,046–66,520 pg/mL, for the median and interquartile range (IQR), respectively, *p* = 0.01) (Figure 1c). The principle concentration of the growth factor found in the human colostrum was dominated by TGF-β2, followed by HGF, TGF-β3, and TGF-β1, respectively. 

The concentration of IgA in the colostrum from Burundian mothers was significantly higher than in the Italian colostrum samples (2.78 and 1.45–22.2 g/L vs. 1.48 and 0.89–2.67 g/L, for the median and interquartile range (IQR), respectively, *p* < 0.01) (Figure 1e). 

#### 3.2.2. Multivariate Analysis Results

The multivariate linear regression analysis was used to adjust for the factors that may affect the immune mediator concentration in human milk. The backwards regression models calculated the most significant factors contributing to the differences in concentration, shown in Table 2. The difference in the TGF-β2 and IgA concentrations between the countries remained significant upon adjustment for potential confounders. The levels of HGF, which were at a borderline significance at the univariate analysis (*p* = 0.08), showed a significant difference upon adjustment.

#### 3.2.3. Correlation of TGF-β1, 2, and 3 with IgA Levels

To assess the correlation between the levels of TGF-β, we used Spearman’s rank correlation coefficients. We found moderate positive correlations between the growth factor and IgA concentrations in the colostrum of Italian mothers (*p* < 0.01) and a moderate to strong correlation in the samples from the Burundian mothers. The growth factors correlated with IgA were apparently stronger in the colostrum samples from Burundi, as seen in Table 3.

## 4. Discussion

This study aimed to evaluate the differences in the growth factors (TGF-β isoforms and HGF) and IgA levels in the colostrum of women from two distinct cohorts, West African and a Southern European. A great variability in the microbial composition, risk of severe infections, and non-communicable disease rates is seen in these two populations. We found a few apparent differences between the sites following the unadjusted and adjusted analysis. The raw concentrations assessment identified significantly higher concentrations of TGF-β2 and IgA in the Burundian samples and a cursory trend for the HGF concentration in the colostrum samples from Burundi, which reached statistical significance upon adjustment. 

Our results showed no significant difference in the TGF-β1 and 3 concentrations between the two groups of women. In contrast, a few studies previously found higher levels of TGF-β1 in the human milk of mothers from developing countries [27,37], or from those residing in a farming environment [38]. The authors suggested that maternal contact with high microbial environments was attributed to an increase of TGF-β1. As multiple factors may influence the immune marker concentration in human milk, it can be speculated that the differences between the studies are a result of ethnic and dietary differences. A major limitation of all of the abovementioned studies, as well as our own work, is a limited sample size. To fully understand the cause of the TGF-β1 variations, the assessment of the dietary and genetic profiles of mothers from different geographical locations is required.

We found higher concentrations of TGF-β2, the predominant isoform of TGF-β in human milk, and IgA in the Burundian colostrum samples. Existing differences in concentration between the sites were confirmed upon adjustment for potential confounders. This finding is in agreement with the results of the study comparing human milk samples from Estonia and Sweden, with higher secretory IgA levels found in Estonia, a country with a greater microbial burden [39]. The high bacterial and viral load in Western Africa may trigger the maternal immune responses and amplify the production of immune mediators; this induces in utero epigenetic alterations via placental Fc receptors [40]. Postnatally, the maternal immune mediators are passed to the infant through breast milk. Thus, the increased TGF-β2 and IgA concentration in the colostrum reflects their purpose, to target microbial antigens and improve mucosal barrier function [41,42]. It may be hypothesised that the elevated IgA concentrations are a potential ‘compensation’ for the high microbial burden in Burundi. 

The surroundings in sub-Saharan Africa offer a microbial load and may possibly stimulate the elevated HGF concentration that was found in colostrum from the Burundian mothers, as a result of the immune-protective effects of HGF on the newborn mucosal barriers [11]. 

The gestational age of the Burundian infants was lower than their Italian peers. This could potentially result in a greater immunological need, and the mothers’ milk is more likely to resemble a preterm milk composition, with higher levels of IgA and TGFβ2 [43], which could be another plausible explanation for the increased levels of these markers in the Burundian women’s milk.

In Italy, a more industrialised country with fewer microbial exposures, there is decreased maternal immune stimulation and we detected lower levels of TGF-β2 and IgA in the colostrum of the Italian women. This is supported by the ‘hygiene hypothesis’, which associated Western lifestyles with rising non-communicable disease prevalence as a consequence of decreased microbial exposures in childhood [44]. Previously, Hawkes and colleagues established that the maternal plasma concentrations of TGF-β2 do not correlate to the levels found in paired breast milk samples [45]. It was also suggested that throughout pregnancy and lactation, the immune cells infiltrate the mammary tissue to upregulate the production of key soluble factors, including TGF-β2 [46]. These studies propose that the local mammary gland production of TGF-β2 may be responsible for the high concentrations seen in breast milk. Therefore, our results cannot unambiguously confirm that systemic immune responses due to a higher maternal microbial exposure in Burundi are a causal factor for the differences in the TGF-β2 levels between the sites. Population studies and genome wide analysis can be used to assess the relationship of host–pathogen responses in human milk components between the countries. However, our results provide additional data, improving the understanding of the selective pressures that lead to immune component variability in human milk and the factors responsible for the differences observed. 

The levels of each of the TGF-β isoforms significantly correlated with the IgA concentration in the colostrum from both Burundi and Italy, with stronger correlations observed in the colostrum of the Burundi women. Correlations of immunological markers in human milk are a well-known phenomenon [47], however, it is worth noting that the strength of the correlation in different geographical locations is rarely assessed and should be considered as a potential topic of interest in the future. TGF-β is known to mediate the class-switching of antibodies and therefore regulates the production of secretory IgA [48]. Some authors have suggested that additional signaling factors are required for complete IgA differentiation and class-switching, such as interleukin (IL)-2 and IL-5 [49,50]. We are far from completely understanding the IgA production trigger, but it is necessary to consider the contributing signals for antibody class-switching. This is a possible area that may be considered for further investigation. 

Understanding the immune mediator composition of human milk and its’ relationships with communicable and non-communicable disease progression is an area of great interest, as it could lead to the development of various therapeutic applications. This includes manipulation with the maternal diet or the environment so as to promote ‘healthier’ human milk composition, as well as the fortification of formula milk for those infants unable to receive breast milk. Some animal model studies show promising outcomes [51], and there are targets for application in humans [34]. It is widely believed that airborne allergens in human milk can be used to induce TGF-β1 mediated oral tolerance [52]. This may be a possible intervention to prevent antigen-specific allergic airway diseases, but also may have applications in infectious diseases. By understanding the transfer of maternal plasma immune mediators to breast milk, there is vast potential for the development of vaccines that could immunise both mother and infant [53]. 

With the emergence of data showing that the differences in human milk immune composition most likely exist between the countries [26,29,38,39,54], it becomes more apparent that it should be considered as an important confounding factor in breastfeeding research. At present, most of the studies evaluating the associations between breastfeeding and health outcomes do not take differences in the breast milk composition into account, which may lead to a serious bias. This becomes particularly important when the data from the developed and developing world is compared. The diversity of the human milk composition may account for the conflicting data coming from breastfeeding studies.

More intense research of the differences between the human milk immunological composition may result in an improvement of donor human milk routine use approaches. This is particularly important for a high-income, well developed countries, when necrotizing enterocolitis (NEC) is concerned [55]. A recently published Cochrane systematic review suggests that infant formula use in preterm infants results in an almost doubled risk of NEC development, when compared with donor human milk [56]. A better understanding of human milk immune composition may help to highlight the important markers that are able to further reduce the risk of NEC development and select the most appropriate milk for the infant at risk.

Our study had several limitations; the maternal dietary patterns have not been assessed using a food frequency questionnaire and the maternal body mass index (BMI) has not been measured; we did not evaluate the maternal genotype as a potentially important determinant or modulator of human milk composition; and the small cohort size from Burundi represented another limitation within our study. Although a larger number of samples were collected from the Burundi mothers, because of missing data from the mother–newborn pairs, some data were excluded from the statistical analysis, which led to a reduced statistical power. A small sample size could potentially skew the outcomes obtained on a multivariate linear regression analysis. We sampled single colostrum specimens from each subject, which can also be considered as a limitation. The time before freezing has not been recorded, but it is extremely unlikely to have influenced the results as the samples were frozen within a few hours of collection, and it has been previously shown that the immune active molecules are very stable and do not degrade rapidly [57,58]. The difference in the initial storage conditions (−20 °C and −50 °C), for a period of less than 12 months, is also very unlikely to have had a significant effect on the immunological composition [58]. The outcomes of the statistical analysis are somewhat promising, as the univariate analysis results were subsequently confirmed when the multivariate testing was applied. Another advantage of this study is the rigorous standardisation of the sample collection and analysis, as well as the measurement of important immunological markers, using a very sensitive technique. 

## 5. Conclusions

This cross-sectional study has found differences in the colostrum immune composition between women residing in Burundi and Italy, with higher levels and stronger correlations between the markers found in the milk of the African women. Taken together, these results suggest that differences in the environment and a higher bacterial upload in the developing countries may lead to a higher excretion of factors in to the human milk, in order to provide better protection against infections, as a compensatory mechanism. A stronger correlation between the growth factors and the IgA in colostrum of the women residing in Africa needs further investigation. 

The results of this research support the idea that maternal and environmental differences between the populations may result in human milk compositional changes. However, the generalisability of these results is subject to certain limitations. For instance, a small sample size does not allow us to make definitive conclusions. What is now needed is a cross-national study involving participants from very diverse geographical locations, in order to fully determine the cause of the differences in the colostrum composition, taking into account multiple factors that may influence the outcomes. 

## Figures and Tables

**Table 1:** Characteristics of study participants between sites of collection.

Characteristics	Burundi	Italy	*p*-Value
Maternal age (years), mean (SD) ^1^	24.30 (5.57)	37.39 (5.38)	**<0.01**
Birth weight (grams), mean (SD) ^1^	2831 (746.8)	3328 (476.9)	**<0.01**
Gestational age (weeks), mean (SD) ^1^	36.48 (1.12)	39.36 (1/34)	**<0.01**
Gender (male), *n* (%) ^2^	15/25 (60)	41/76 (54)	0.60
Mode of delivery (c-section), *n* (%) ^3^	7/21 (33)	14/76 (18)	0.14
Parity, mean (SD) ^4^	2.04 (1.15)	0.81 (0.80)	**<0.01**
Maternal smoking, *n* (%) ^5^	2/23 (9)	3/76 (4)	0.33
Antenatal infections ^6^	6/23 (26)	29/76 (38)	0.29
Regular animal contact ^6^	10/23 (43)	27/76 (36)	0.49
Time of colostrum collection (hours), mean (SD) ^1^	58.29 (26.4)	51.13 (32.61)	0.28

Statistical methods used: ^1^ Unpaired *t* tests. ^2^ Pearson chi-squared and data dichotomised into groups of female and male. ^3^ Pearson chi-squared test and data dichotomised into groups of vaginal delivery and caesarean section (c-section) delivery. ^4^ Mann–Whitney U test. ^5^ Fisher’s exact test. ^6^ Pearson chi-squared test. Statistically significant results presented in bold. SD—standard deviation.

**Table 2:** Univariate and adjusted analysis for comparison of growth factors and IgA levels in the colostrum of women from Burundi and Italy.

Immune Factor	Burundi Median (IQR)	Italy Median (IQR)	Unadjusted *p*-Values ^1^	Adjusted Analysis ^2^	
				Most Important Factor ^3^	*p*-Value
HGF (pg/mL)	2785 (697.4–10,107)	1316 (785–2628)	0.08	Country of residence	<0.01
TGFβ1 (pg/mL)	673.1 (387.5–1133)	663 (483.1–940.4)	0.80	Parity	0.13
TGFβ2 (pg/mL)	59,708 (36,865–113,221)	33,176 (18,046–66,520)	0.01	Country of residence	0.03
TGFβ3 (pg/mL)	1056 (456.5–3212)	1066 (566.3–2038)	0.94	Parity	0.11
IgA (g/L)	2.78 (1.45–22.2)	1.48 (0.89–2.67)	<0.01	Country of residence	<0.01

^1^ Statistical analysis for unadjusted comparisons between women from Italy and Burundi, using Mann–Whitney U test; ^2^ adjusted analysis (multivariate linear regression). Confounding factors included in the model: country of residence, parity (binary), maternal age, gestational age, and animal contact during pregnancy; ^3^ According to multivariate regression model; Statistically significant results (at *p* value < 0.05) presented in bold. interquartile range—IQR; TGF-β—transforming growth factor-β; HGF—hepatocyte growth factor; IgA—immunoglobulin A.

**Table 3:** Correlation between levels of growth factors (TGFβ1, 2, and 3) with IgA.

Correlating Factors	Burundi (*r* ^1^)	Italy (*r*)
HGF/IgA	0.71 **	0.38 **
TGF-β1/IgA	0.52 *	0.28 *
TGF-β2/IgA	0.68 **	0.32 **
TGF-β3/IgA	0.72 **	0.51 **

Statistical analysis was performed using Spearman’s rank correlation coefficient. ^1^ Spearman *r* values are presented; *—results are statistically significant at a level less than 0.05; **—results are statistically significant at a level less than 0.01.
